# Role of cerebral cortex in the neuropathology of Huntington's disease

**DOI:** 10.3389/fncir.2013.00019

**Published:** 2013-02-18

**Authors:** Ana M. Estrada-Sánchez, George V. Rebec

**Affiliations:** Program in Neuroscience and Department of Psychological and Brain Sciences, Indiana UniversityBloomington, IN, USA

**Keywords:** basal ganglia, glutamate transmission, huntingtin, neuronal processing

## Abstract

An expansion of glutamine repeats in the N-terminal domain of the huntingtin protein leads to Huntington's disease (HD), a neurodegenerative condition characterized by the presence of involuntary movements, dementia, and psychiatric disturbances. Evaluation of postmortem HD tissue indicates that the most prominent cell loss occurs in cerebral cortex and striatum, forebrain regions in which cortical pyramidal neurons (CPNs) and striatal medium spiny neurons (MSNs) are the most affected. Subsequent evidence obtained from HD patients and especially from transgenic mouse models of HD indicates that long before neuronal death, patterns of communication between CPNs and MSNs become dysfunctional. In fact, electrophysiological signaling in transgenic HD mice is altered even before the appearance of the HD behavioral phenotype, suggesting that dysfunctional cortical input to the striatum sets the stage for the emergence of HD neurological signs. Striatal MSNs, moreover, project back to cortex via multi-synaptic connections, allowing for even further disruptions in cortical processing. An effective therapeutic strategy for HD, therefore, may lie in understanding the synaptic mechanisms by which it dysregulates the corticostriatal system. Here, we review literature evaluating the molecular, morphological, and physiological alterations in the cerebral cortex, a key component of brain circuitry controlling motor behavior, as they occur in both patients and transgenic HD models.

## Introduction

In 1872, George Huntington described a disease that affects certain families and is characterized by the presence of choreic movements that gradually increase in severity and variety; his description led to the name Huntington's disease (HD; Huntington, 1872, 2003). Although Huntington described the inherited feature of HD, it was not until 1983 that a DNA marker linked to HD was identified at the tip of chromosome 4 (Gusella et al., 1983). A decade later, in 1993, the same research group reported that the HD mutation consists of an unstable CAG repeat in exon 1 of the gene that codes for the protein called “huntingtin” (HTT; HD Collaborative Research Group, 1993). Normally, this exon contains between 3 and 30 CAGs, but if the number of CAGs exceeds 35, HD becomes increasingly likely with full penetrance occurring at ≥39 repeat (Harper, 2001). A rough inverse correlation between the number of CAGs and the age of HD onset occur (For review see, Langbehn et al., 2010). While individuals carrying 40 CAG repeats are likely to show the first signs of HD at 35–40 years of age; CAG repeats ranging from 50 to 200 (the highest number reported) may precipitate HD onset in childhood or teenage years. The last is characterized with psychiatric disturbances, accelerated mental and physical deterioration that culminates in death as soon as 5–10 years after onset (Rasmussen et al., 2000; Quarrell et al., 2012).

In adult-onset HD, patients show a progressive deterioration of motor and cognitive function that follows three well-defined stages spread over 15–20 years (Harper, 1991). At the initial stage and before the appearance of prominent motor alterations, HD patients are likely to show psychiatric symptoms that include apathy, irritability, depression, and other mood alterations (Harper, 1991; Reedeker et al., 2012; Thompson et al., 2012). Slight motor abnormalities, such as motor tics and jerky voluntary movements, also are likely (Beste et al., 2009). The second stage is characterized by a dramatic increase in involuntary movements, which become generalized, abrupt, and uncontrolled. As these choreic movements become more prominent, daily activities such as walking, eating, speaking, and swallowing deteriorate. In some cases, bradykinesia may coexist with the choreic movements that become prominent in the third stage (Thompson et al., 1988). Cognitive capacities progressively decline and finally culminate in dementia. Another hallmark of the second stage is the loss of body weight despite efforts to maintain a high caloric diet (Marder et al., 2009). Overall health progressively deteriorates and by the third stage, which typically occurs 10–15 years after diagnosis. In this stage, choreic movements are replaced by bradykinesia and rigidity. Death becomes imminent, and the most common causes are pneumonia and heart disease.

Soon after the HD gene was identified, different transgenic animal models were developed. The models, together with studies performed on HD patients and on postmortem HD brains, revealed that although mutant HTT is ubiquitously expressed, the most prominent pathology occurs in cerebral cortex and striatum. Cortical pyramidal neurons (CPNs) send massive and widespread projections to the striatum, forming the corticostriatal pathway that among other functions shapes motor behavior. Because the development of involuntary movements is a prominent feature of the HD phenotype, dysfunctional cortical input to striatum is likely to constitute a key component of HD neuropathology. In fact, emerging evidence from transgenic HD models suggests that the expression of mutant HTT alters the pattern of communication between CPNs and medium spiny neurons (MSNs) in the striatum. Striatal MSNs, moreover, send information to downstream structures that project to thalamus, which in turn projects back to cortex, allowing for even further disruptions in cortical processing. Here, our aim is to review the functional alterations in cerebral cortex in both patients and transgenic models of HD and to assess how these alterations disrupt the corticostriatal system to drive the HD phenotype.

## Overview of cerebral cortex organization

The cerebral cortex is the external sheet of neuronal tissue that covers the entire brain. In mammals, cerebral cortex folds together forming the “sulci” which compress a large portion of cortical tissue in a small area. In humans the thickness of the cerebral cortex is subject to regular local variations, but the average values range from 1.5 to 4.5 mm (Brodmann, 2010). The cortical mantle is divided into specialized regions that control language, decision-making, and motor activity, among other functions. Cortical neurons are organized into six main layers; the most superficial lamina (Layer I) contains apical dendritic tufts of CPNs and horizontal axons; it might also include the so-called Cajal-Retzius and spiny stellate cells (von Economo, 1927). Layer II is composed of numerous small granule cells. Layer III, contains large and robust CPNs, along with interneurons (von Economo, 1927; Ichinohe, 2012). Layer IV, consists of densely packed small polymorphous granule cells. Layer V, contains large CPNs. Layer VI, contains large CPNs and small spindle-like pyramidal and multiform neurons (von Economo, 1927). In addition to the different neuronal populations, astrocytes are homogeneously distributed throughout the six-layered cortical organization (von Economo, 1927).

Cortical neurons make local connections between cells of different layers, also send and receive projections from other brain regions (Szentágothai, 1975). For example, layer I in visual cortex receives axonal ramifications from apical dendrites of pyramidal neurons located in layers III and IV (Shipp, 2005, 2007). Layer I also receives input from thalamic (matrix) M-type neurons (Rubio-Garrido et al., 2009). Lateral connections occur between neurons in layers II and III, but these neurons also send projections to layer **V** (Shipp, 2007). Neurons located in layer IV project to layers II and III (Miller, 2003). In somatosensory cortex, pyramidal neurons in layer IV are the main target of thalamocortical afferents from type (core) C-neurons (Jones and Pons, 1998). CPNs from layer V are the principal source of input to the basal ganglia, brain stem, and spinal cord. CPNs in layer VI send projections to thalamus (Shipp, 2007). Although similar intrinsic and extrinsic connections are thought to occur along the length of the cortical mantle, significant deviations occur among some functionally distinct cortical areas. Detailed descriptions of cortical connections are available elsewhere (Sherman and Guillery, 2011; Espinosa and Stryker, 2012; Nieuwenhuys, 2012; Ray and Zald, 2012). In the next section, we focus on cortical motor areas and their projections to basal ganglia.

## Motor cortex and its projections to basal ganglia

Motor cortex refers to the cortical areas associated with the planning, execution and control of voluntary movements. Motor cortex is subdivided into multiple motor areas: primary motor cortex (M1), premotor cortex dorsal caudal (PMdc), premotor cortex dorsal rostral (PMdr), cingulate motor area caudal (CGc), cingulate motor area rostral (CGr), premotor cortex ventral caudal (PMvc), premotor cortex ventral rostral (PMvr), pre-supplementary motor area (Pre-SMA), and supplementary motor area (SMA; Schieber and Baker, 2003). These areas were identified by evaluating motor responses elicited by electrical stimulation in different species, including humans (Foerster, 1936; Penfield and Boldrey, 1937; Bures and Bracha, 1990). Thus, motor areas are localized to Brodmann's cortical areas 4 (the giant pyramidal area) and 6 (agranular frontal area) (Brodmann, 2010). Brodmann's areas 23 and 24 are now known to contain at least two additional motor areas (Schieber and Baker, 2003). Motor cortical neurons follow the laminar organization, but motor cortex lacks granular layer IV and layer III is relatively thinner (Brodmann, 2010). Motor cortex also is characterized by the presence of giant pyramidal neurons known as Betz cells located in layer V; these cells are the largest neurons in the brain, reaching dimensions of 60–120/30–80 μm: height/width (von Economo, 1927; Rivara et al., 2003).

Motor cortical projections to the striatum, the first information processing stage of the basal ganglia (Jinnai and Matsuda, 1979; McGeorge and Faull, 1989), arise mainly from layer V, although retrograde labeling studies also identify pyramidal projections from layer III. Cortical projections are bilateral with an ipsilateral predominance and release glutamate, an excitatory amino acid transmitter (McGeorge and Faull, 1989). Besides motor cortical areas, the striatum receives projections form sensory and associative cortical areas (Kemp and Powell, 1970). Two types of striatal-projecting CPNs have been identified. They can be distinguished by the morphology of their projection: pyramidal tract (PT)-type neurons project to the striatum via collaterals arising from the main axon that extends to the PT and intra-telencephalically projecting (IT)-type neurons that project to striatum and cortex but not to areas outside telencephalon (Lévesque et al., 1996; Reiner et al., 2003). Anatomical and functional differences have been described between PT- and IT- type neurons (For review see, Reiner et al., 2010). While the PT type is mainly found in lower cortical layer V, the IT type is located in layer III and upper layer V (Reiner et al., 2003; Parent and Parent, 2006). Likewise, PT- and IT-type neurons differentially target striatal MSNs such that IT-type neurons preferentially target MSNs of the direct pathway and PT-type neurons preferentially innervate MSNs of the indirect pathway (Lei et al., 2004; Reiner et al., 2010). MSNs are GABAergic neurons that constitute 95% of the neuronal population of the striatum and comprise its main output system. Two MSN subtypes have been identified according to the proteins they express and their synaptic targets. MSNs that preferentially express substance P, dynorphin, and the D1-like dopamine receptor make up the direct pathway, which projects to the internal segment of the globus pallidus (GPi) and substantia nigra pars reticulata (SNr). The indirect pathway is composed of MSNs that contain enkephalin and express the D2-like dopamine receptor; these neurons project to the external segment of the globus pallidus (GPe) and the subthalamic nucleus, while also sending collaterals to GPi and SNr (Smith et al., 1998).

Understanding the motor neuronal pathways involved in control, planning and execution of motor behavior is primary for understanding neurophatologic conditions such as HD, in which one of the main phenotypic alterations is the development of involuntary movements.

## Cerebral cortex in HD: postmortem studies

Postmortem evaluation of the human HD brain indicates a 30% reduction of total brain weight (De la Monte et al., 1988). The most striking feature is neuronal loss in striatum (caudate-putamen) along with enlarged lateral ventricles (De la Monte et al., 1988). A scale of neuropathology based on the extent of striatal atrophy has been developed (Vonsattel et al., 1985). According to this scale, Grade 0 indicates that clinical symptoms are noticeable but with no observable neuropathological alterations. Grade 1 is characterized by no macroscopic evidence of damage but some neuronal loss and the presence of gliosis. Progressive striatal atrophy is observed in Grades 2 and 3, which includes substantial reductions in the neuronal population (>50%) and increased gliosis. Marked striatal atrophy (95% neuronal loss) and widespread gliosis define Grade 4. In addition to striatal damage, cortical changes also have been observed in postmortem studies of HD patients. Reduced cross-sectional area of gray and white matter has been reported for frontal, temporal, insular, and parietal cortical areas in HD Grades 2–4 (Mann et al., 1993; Halliday et al., 1998). Likewise, De la Monte et al. (1988) described a progressive reduction in overall cortical area and in cortical white matter during mild (Grades 1 and 2) and severe HD (Grades 3 and 4). There also is evidence of reduced thickness of the cortical ribbon (De la Monte et al., 1988; Heinsen et al., 1994). These findings are consistent with a 30% reduction in the number of CPNs in cortical layer III, V and VI (Cudkowicz and Kowall, 1990; Hedreen et al., 1991; Sotrel et al., 1991; Heinsen et al., 1994). Moreover, primary motor cortex (Brodmann's area 4) and the premotor area (Brodmann's area 6) of HD brains show a similar reduction in CPN numbers (Macdonald and Halliday, 2002; Thu et al., 2010). Interestingly, the extent of cortical atrophy in motor areas roughly correlates with the extent of the HD motor phenotype (Halliday et al., 1998; Thu et al., 2010). It is equally striking that in HD patients for whom mood alterations are the primary symptom, the loss of CPNs is most prominent in cingulate cortex, which processes emotion (Thu et al., 2010). On the other hand, despite progressive loss of CPNs, postmortem studies indicate that parvalbumin-, calretinin- and calbindin- positive interneurons appear to be normally distributed without evident morphological changes in motor cortical areas (Macdonald and Halliday, 2002).

Together, these postmortem assessments indicate that cortical atrophy and loss of CPNs is a neuropathological hallmark of HD. Most of these studies, however, were performed on tissue from neuropathological grades 2–4; little is known about cortical atrophy at early HD stages and even before symptoms develop. More importantly, a growing body of evidence from both HD patients and transgenic models suggests that alterations in neuronal communication precede neuronal loss and play a critical role in the development of HD symptoms. Early support for this view emerged from evaluation of proteins associated with synaptic transmission, including receptors, transporters, intra-cellular signaling pathways, and vesicular neurotransmitter release (Cross et al., 1986; Dunlop et al., 1992; Arzberger et al., 1997; Smith et al., 2007; Hassel et al., 2008; Faideau et al., 2010). Subsequent support has come from neuroimaging studies and recording of neuronal activity (Miller and Bezprozvanny, 2010; Estrada-Sánchez and Rebec, 2012; Hong et al., 2012c; Unschuld et al., 2012). The following sections focus on the cellular dysfunctions that are the likely drivers of the HD behavioral phenotype.

## Functional alterations in cerebral cortex: HD imaging studies

Neuroimaging studies permit visualization of pathological changes *in vivo*, before clinical symptoms develop. Techniques such as magnetic resonance imaging (MRI), positron emission tomography (PET), and single-photon-emission computed tomography (SPECT) have been used to detect brain atrophy and functional changes in pre-symptomatic HD patients. These approaches have not only confirmed the thinning of cerebral cortex, but also showed that this effect occurs early, develops progressively, and extends through the cortical mantle, although distinct grades of cortical thinning may occur (Rosas et al., 2002, 2008, 2011). Interestingly, patterns of cortical thinning are associated with both the cognitive and motor phenotypes. For example, patients with more prominent bradykinesia and dystonia show more significant thinning in premotor and supplementary motor areas (Rosas et al., 2008). Moreover, longitudinal MRI evaluations indicate that the progression and topological distribution of cortical thinning is influenced by age of onset, suggesting an important role of cerebral cortex in HD neuropathology (Rosas et al., 2011).

Besides morphological changes, cerebral cortex also undergoes several changes that might compromise neuronal communication. SPECT, a technique based on alterations in blood flow, indicates reduced cerebral blood perfusion in presymptomatic HD patients (Hasselbalch et al., 1992; Sax et al., 1996). Reduced blood flow is widely observed along the cortical mantle and precedes the presence of cortical atrophy as evaluated by MRI (Jernigan et al., 1991; Hasselbalch et al., 1992; Sax et al., 1996). Given that blood provides oxygen and glucose essential for brain function, reduced blood perfusion in HD is likely to compromise neuronal signaling mechanisms. Accordingly, reduced glucose uptake and glucose metabolism have been identified in striatum and cerebral cortex of pre-symptomatic HD patients, as studied by 18F-fluorodeoxyglucose and PET (Kuhl et al., 1982; Kuwert et al., 1989, 1990; Jenkins et al., 1998). The rate of glucose uptake and its metabolism is an indicator of altered neuronal activity. Thus, these studies suggest altered cortical function before HD symptoms become prominent, an effect that could alter communication between CPNs and deep brain structures. In fact, prodromal-HD patients show reduced corticostriatal functional connectivity (Unschuld et al., 2012). Consistent with this view, neuroimaging studies indicate white matter atrophy before the onset of clinical symptoms (Fennema-Notestine et al., 2004; Rosas et al., 2006, 2010). It appears, therefore, that compromised cortical circuitry occurs early in the course of HD (Rosas et al., 2006).

Collectively, these studies indicate that in HD the cerebral cortex undergoes morphological and functional changes that occur early in disease progression. Combined with evidence that the cortical changes parallel the manifestation of both cognitive and motor symptoms, these studies underscore the key role of cortical neuropathology in HD. Neuroimaging studies, moreover, suggest that cortical dysfunction rather than neuronal loss is a key factor in HD onset. This hypothesis gained considerable support from research on transgenic HD mice, which is reviewed in the next sections.

## Transgenic HD models

Transgenic mouse models of HD are often separated into three broad categories based on the genetic manipulation: truncated, full-length, and knock-in models. The truncated model expresses only the first exon of the human mutant *htt* gene, while the full-length model expresses the entire human mutant *htt* gene. Thus, these two models express two normal alleles of the endogenous *Hdh* gene (the murine analog of *htt*) along with all or part of the exogenous human *htt* gene. In the knock-in model, CAG repeats are “knocked in” or directly inserted into the first exon of the endogenous *Hdh* gene.

Truncated models were developed first, and are represented by the R6 line of mice. Two variants are available: the R6/1 line, which contains ~110 CAG repeats, and the R6/2 line, which contains ~150 CAG repeats (Mangiarini et al., 1996). More recently, other truncated models have been created, including the N171-82Q mouse that expresses cDNA encoding 171 amino acids in the first exon of the *htt* gene with 82 CAG repeats (Schilling et al., 1999). A truncated transgenic HD rat model with 51 CAG repeats also has been created (von Hörsten et al., 2003). Like the truncated R6 line, the full-length HD model has two variants: the yeast artificial chromosome (YAC) and the bacterial artificial chromosome (BAC) models, the names of which refer to the genetic tool (yeast or bacteria) used to insert the human mutant *htt* gene. Three different YAC models are available based on CAG-repeat length, YAC46, YAC72, and YAC128; the latter is the most widely studied of this group (Hodgson et al., 1999). The BAC model expresses 97 CAG repeats (Gray et al., 2008). Finally, several knock-in variants have been developed, including: *Hdh*/Q72-80, *Hdh* Q111, CAG140, and CAG150 models, again with the numbers indicating CAG-repeat length (Menalled, 2005).

Despite the genetic difference that exists among HD transgenic models, they all develop a form of the behavioral phenotype and neuropathology seen in patients. HD transgenic models however, show varying degrees of behavior and neuropathological alterations. Truncated R6 models, for example, develop accelerated progressive motor alterations, reduced body weight, and shortened lifespan (~10–14 and 3–5 months for R6/1 and R6/2, respectively) (Mangiarini et al., 1996). At the neuropathological level, these models show intracellular and intranuclear aggregates containing the mutant HTT fragment and other proteins; reduced striatal volume and neuronal atrophy also occur (Mangiarini et al., 1996; Davies et al., 1997; Stack et al., 2005). The N171-82Q model also has decreased body weight and motor alterations, but these are less severe than in the R6/2 model (Kim et al., 2011). The neuropathological changes, however, are similar to those described in the R6 models (Yu et al., 2003; McBride et al., 2006; Kim et al., 2011). In the case of the truncated HD transgenic rat, there are intracellular aggregates, but unlike the truncated mouse model, neurological deficits emerge in adulthood along with progressive motor alterations (von Hörsten et al., 2003). Full length models, on the other hand, show important differences relative to truncated models. For example, a notable increase in body weight occurs in the BACHD and YAC128 models (Van Raamsdonk et al., 2006; Pouladi et al., 2010, 2012). Both also show reduced striatal volume, although the presence of intracellular aggregates is more prominent in the striatum of YAC128 models (Gray et al., 2008; Spampanato et al., 2008; Miller et al., 2011b; Pouladi et al., 2012). Although full length models show impaired performance in motor tests, the motor changes are relatively mild compared with truncated models (Ferrante, 2009; Kim et al., 2011). Similar to full length models, knockin HD mice show slowly emerging motor changes along with intracellular aggregates and striatal atrophy (For review see, Menalled, 2005). Interestingly, the neuropathology in aged knockin mice (21 months of age) resembles that described for R6/2 mice, including widespread expression of aggregates and transcriptional alterations (Woodman et al., 2007).

These findings indicate important differences in the neural and behavioral phenotypes among the HD models. However, it is important to note that despite these differences, behavioral alterations appear prior to neuronal loss, suggesting that HD neurological signs are likely to be caused by impaired neuronal communication, particularly in the corticostriatal pathway.

## Dysfunctional corticostriatal network in HD; evidence from transgenic models

Electrophysiological recordings of CPNs in truncated and full length HD models indicate a change in electrical properties such as resting membrane potential, input resistance, and cell capacitance (Cummings et al., 2006, 2009). Similar membrane changes were observed *in vivo* during intracellular recordings of R6/2 CPNs (Stern, 2011). Likewise, there is consistent evidence of an imbalance between excitatory and inhibitory inputs to CPNs in HD, which may underlie hyperexcitable cortical neurons reported for HD models (Gu et al., 2005; Spampanato et al., 2008; Cummings et al., 2009).

Impaired CPN activity in HD also is likely to interfere with synaptic plasticity. Progressive alterations in long-term depression (LTD) occur in the perirhinal cortex of R6/1 mice (Cummings et al., 2006, 2007). Similarly, CPNs in medial prefrontal cortex show impaired long-term potentiation (LTP) (Dallérac et al., 2011). Interestingly, in both cases, the loss of synaptic plasticity can be reversed by the activation of dopamine receptors (Cummings et al., 2006, 2007; Dallérac et al., 2011), suggesting an HD-induced change in dopamine modulation of cortical processing. This change may underlie the deficient cortical plasticity observed in R6/1 model performance in a somatosensory-discrimination learning task (Cybulska-Klosowicz et al., 2004; Mazarakis et al., 2005).

Dysfunctional interneurons also may contribute to impaired CPN activity in HD. For example, reduced GABAergic inhibitory input from interneurons occurs in HD models (Gu et al., 2005; Cummings et al., 2009). In BACHD mice at 3 months of age—when the motor phenotype is relatively mild—parvalbumin-containing interneurons in layers II and III show alterations in the kinetics of decay of spontaneously occurring inhibitory and excitatory postsynaptic currents (sIPSC and sEPSC, respectively; Spampanato et al., 2008). At 6 months, when motor alterations are more prominent, parvalbumin-containing interneurons show decreased excitation, while CPNs show decreased inhibition, indicating increased cortical excitability (Spampanato et al., 2008). Thus, cortical interneurons and CPNs show alterations that parallel the progressive motor phenotype seen in the BACHD model. Moreover, these results also suggest that interneurons might have an important role in the HD phenotype, given that their alterations may precede CPN dysfunction. In line with this view, it has been suggested that inhibitory interneurons are involved in shaping motor commands (For review see, Merchant et al., 2012).

Neurons communicate through the modulation of firing rate and by the generation of spike bursts, which are epochs of high firing frequency. Burst firing is relevant for the efficiency of neuronal transmission and also plays a role in neuronal plasticity (Lisman, 1997; Izhikevich et al., 2003). Cortical activity recorded in freely behaving R6/2 mice is characterized by decreased bursting activity and a decrease in the number of spikes that participate in a burst (Walker et al., 2008). Similarly, reduced bursting occurs in cultured cortical neurons expressing a fragment of mutant HTT (Gambazzi et al., 2010). Cortical neurons in both R6/2 and *Hdh* CAG140 mice are more likely to show a decrease in correlated activity between simultaneously recorded pairs of neurons than corresponding wild-type controls (Walker et al., 2008). Consistent with this evidence, *in vivo* intracellular recordings in HD mice indicate a decreased correlation between cortical spiking and simultaneously recorded electrocorticograms relative to wild-type (Stern, 2011). Importantly, correlated neuronal activity is a key factor in the operation of the neuronal circuits that shape behavior (Berke et al., 2004; Buzsáki and Chrobak, 2005; Burns et al., 2010), suggesting that deficient synchronous activity in cortical HD neurons is associated with the behavioral alterations in HD transgenic models (Walker et al., 2011). Interestingly, local field potentials (LFPs), which represent the activity of large populations of neurons in the vicinity of the recording electrode, also are altered in cortex of HD mice (Hong et al., 2012c). In this case, however, LFPs show the greatest deviation from wild-type during quiet rest and less so as behavior switches to grooming and exploring. It may be that the high frequency oscillations (~32 Hz) in resting HD mice prompt motor activation. In fact, HD mice spend less time resting than wild-type (Hong et al., 2012c).

Along with alterations in CPNs activity, striatal MSNs also show altered electrophysiological properties (reviewed by Cepeda et al., 2007). When studied *in vitro*, MSNs show increased excitability as indicated by a depolarized resting membrane potential and enhanced sensitivity to ionotropic glutamate receptor activation (Hodgson et al., 1999; Levine et al., 1999; Cepeda et al., 2001; Klapstein et al., 2001; Laforet et al., 2001; Milnerwood and Raymond, 2007). This evidence is consistent with increased firing in striatal MSNs recorded from behaving, symptomatic R6/2 mice (Rebec et al., 2006). As in cortex, decreased MSN bursting activity is the most common electrophysiological feature reported for HD models (Miller et al., 2008b, 2010, 2011a; Cayzac et al., 2011). As in CPNs, reductions in correlated firing and coincident bursting between simultaneously recorded pairs of MSNs occur in HD striatum (Miller et al., 2008b, 2010, 2011a; Höhn et al., 2011). In addition, striatal LFPs in behaving R6/2s parallel cortical LFPs in that high frequency gamma activity (focused around 32 Hz) predominates during quiet rest (Hong et al., 2012a,b). Interestingly, LFP oscillations recorded in the globus pallidus of HD patients show alterations in theta/alpha (4–12 Hz) and low gamma (35–45) activity (Groiss et al., 2011).

Collectively, these studies indicate that altered neuronal processing occurs in cerebral cortex of HD transgenic models, indicating that CPNs send aberrant information to MSNs. In fact, analysis of simultaneous recordings of LFPs from primary motor cortex and dorsal striatum, which receives major motor cortical input, indicates an overall reduction in the level of coherence or synchrony between these brain regions in R6/2 relative to wild-type mice across a range of behavioral episodes (Hong et al., 2012c). Given that dysfunctional corticostriatal processing occurs before HD signs are noticeable, these results provide further support for the hypothesis that altered neuronal communication is a prerequisite for the HD behavioral phenotype.

## Mechanism involved in disrupted corticostriatal communication in HD

A critical question is how mutant HTT alters corticostriatal neuronal activity. Two mechanisms have been suggested: cell-autonomous toxicity and cell–cell interaction toxicity (Gu et al., 2005, 2007). The first proposes that the expression of mutant HTT itself is enough to alter the key neuronal mechanisms that lead to neuronal dysfunction. In support of this mechanism, *in vitro* and *in vivo* studies indicate that mutant HTT disrupts gene expression, calcium buffering, and mitochondrial function (Choo et al., 2004; Milakovic and Johnson, 2005; Tang et al., 2005; Thomas et al., 2011). The second mechanism proposes that the interaction between different cell populations is critical for the development of HD. Support for this hypothesis includes altered release of brain derived neurotrophic factor (BDNF) from corticostriatal synapses (reviewed by Zuccato and Cattaneo, 2009), impaired glutamate release from cortical projections (Cepeda et al., 2001; Klapstein et al., 2001; Laforet et al., 2001; Milnerwood and Raymond, 2007), and disrupted glutamate uptake by the astrocytes (Nicniocaill et al., 2001; Hassel et al., 2008). Collectively, these studies indicate that both cell-autonomous and cell–cell interaction toxicity are likely to occur in HD. Thus, a combination of both mechanisms may disrupt neuronal communication and promote development of the HD phenotype.

Impaired energy production appears to be a key alteration in HD, as both HD transgenic models and HD patients show alteration in glycolytic and mitochondrial activity (Browne and Beal, 2004; Powers et al., 2007; Verwaest et al., 2011; Cepeda-Prado et al., 2012). Because neurons are highly dependent on ATP production, decreased glucose metabolism may compromise the activity of Na^+^/K^+^ ATPase, a key protein that helps to maintain the ionic gradient across the neuronal membrane (Bonvento et al., 2002). If the gradient is not maintained by this protein, neurons become depolarized and more excitable, which may explain evidence for increased neuronal excitability in transgenic HD models (see above). Deficient energy production and a loss of the ionic gradient, moreover, may compromise the function of glutamate transporters, transmembrane proteins located in neurons and astrocytes. These transporter proteins depend on the Na^+^ gradient generated by the Na^+^/K^+^ ATPase to ensure glutamate clearance from the synapse and extracellular space. It is noteworthy that decreased glutamate uptake, a well-described feature of HD (Nicniocaill et al., 2001; Hassel et al., 2008; Miller et al., 2008a; for review see Estrada-Sánchez and Rebec, 2012), may result, at least in part, from decreased energy production. In this sense, glycolytic inhibition reduced glutamate transporters levels in the striatum of R6/2 model (Estrada-Sánchez et al., 2010). Moreover, compromised glutamate uptake coupled with impaired cellular metabolism may render neurons vulnerable to excitotoxic damage, a mechanism involved with neurodegeneration of MSNs in HD (Estrada-Sánchez et al., 2008, 2009, 2010). Besides, impaired function of glutamate transporters at corticostriatal synapses might compromise the dynamic of synaptic transmission, leading to aberrant neuronal processing (Beurrier et al., 2009). These mechanisms, of course, are likely to occur in conjunction with many other contributing factors, including altered expression and function of synaptic receptors, increased production of reactive oxidative species, diminished neuronal antioxidant capabilities (For reviews of these and other likely factors see: Browne et al., 1997; André et al., 2006; Chen, 2011; Kaplan and Stockwell, 2012).

## Conclusion

An emerging picture of HD neuropathology includes aberrant cortical signaling that impacts striatal output systems and that then affects thalamic input back to cortex. In short, HD is characterized by dysregulated information flow through the cortico-striato-cortical pathway. This problem, moreover, emerges early in the course of HD, even before some neurological signs are present, suggesting a key role in the development and subsequent progression of the HD behavioral phenotype. Further research on alterations in cortical processing in HD, including the contribution of thalamic inputs and cortical interneurons, is emerging as fertile ground for further insight into the neuronal mechanisms underlying HD and for the development of effective therapeutic strategies.

### Conflict of interest statement

The authors declare that the research was conducted in the absence of any commercial or financial relationships that could be construed as a potential conflict of interest.
